# The establishment of a marine focused biorefinery for bioethanol production using seawater and a novel marine yeast strain

**DOI:** 10.1038/s41598-018-30660-x

**Published:** 2018-08-14

**Authors:** Abdelrahman Saleh Zaky, Darren Greetham, Gregory A. Tucker, Chenyu Du

**Affiliations:** 1 School of Applied Sciences, University of Huddersfield, Huddersfield, HD1 3DH UK; 2 School of Biosciences, University of Nottingham, Nottingham, LE12 5RD UK; 3 Department of Microbiology, Faculty of Agriculture, Cairo University, Giza, 12613 Egypt

## Abstract

Current technologies for bioethanol production rely on the use of freshwater for preparing the fermentation media and use yeasts of a terrestrial origin. Life cycle assessment has suggested that between 1,388 to 9,812 litres of freshwater are consumed for every litre of bioethanol produced. Hence, bioethanol is considered a product with a high-water footprint. This paper investigated the use of seawater-based media and a novel marine yeast strain ‘*Saccharomyces cerevisiae* AZ65’ to reduce the water footprint of bioethanol. Results revealed that *S. cerevisiae* AZ65 had a significantly higher osmotic tolerance when compared with the terrestrial reference strain. Using 15-L bioreactors, *S. cerevisiae* AZ65 produced 93.50 g/L ethanol with a yield of 83.33% (of the theoretical yield) and a maximum productivity of 2.49 g/L/h when using seawater-YPD media. This approach was successfully applied using an industrial fermentation substrate (sugarcane molasses). *S. cerevisiae* AZ65 produced 52.23 g/L ethanol using molasses media prepared in seawater with a yield of 73.80% (of the theoretical yield) and a maximum productivity of 1.43 g/L/h. These results demonstrated that seawater can substitute freshwater for bioethanol production without compromising production efficiency. Results also revealed that marine yeast is a potential candidate for use in the bioethanol industry especially when using seawater or high salt based fermentation media.

## Introduction

An ever-growing population and shifting demographics have led to a continuous increase in global demand for energy. Total world energy consumption is predicted to rise from 549 quadrillion British thermal units (Btu) in 2012 to 815 quadrillion Btu in 2040^1^. Therefore, world CO_2_ emissions related to energy will rise from 32.20 billion metric tons in 2012 to around 43.20 billion metric tons in 2040 with an increase of 34% over the projected period^1^. The Inter-Governmental Panel on Climate Change (IPCC) has reported that, among greenhouse gases, CO_2_ accounts for nearly 55% of the global warming, and therefore, reduction of CO_2_ emissions from fossil fuels is an urgent issue to reduce the global warming trend^2^. Increasing awareness of global warming, climate change combined with petrol price rises has led to the search for alternative sustainable sources of energy. Bioethanol has been considered one of the best fuel alternatives because it is a liquid fuel and has similar characteristics to petrol. Hence, governments in many countries have implemented policies to increase the percentage of bioethanol in their fuel mixes. These policies have promoted a three-fold increase in bioethanol production over the past decade (2000–2010)^3^.

The Agriculture Organization of the United Nations (FAO) projected that global ethanol production will increase from about 120 billion L in 2016 to nearly 137 billion L by 2026^4^. However, the water footprint (WF) of bioethanol ranges from 1,388 to 9,812 litres of water for each litre of ethanol produced; with the majority of the water used in the cultivation of the substrate crops^5^. With the increasing concern about water shortage, water consumption could be a potential barrier for the expansion of bioethanol production. Gerbens-Leenes and Hoekstra concluded that global freshwater resources are limited and allocation of water for bioethanol production on a large scale will be at the cost of water allocation for food and other usages. Therefore, the water usage issue could soon be included in the food and land usage debate^6^.

Several approaches could be applied to reduce water footprint of bioethanol including the use of marine biomass instead of land based biomass and the replacement of freshwater with seawater in the fermentation process^7,8^. Seawater accounts for about 97% of the world’s water and covers approximately 71% of the world’s surface. It is a renewable water source and readily accessible in many countries around the world. Hence, the use of seawater for preparing the fermentation media could be an attractive approach for bioethanol production^7^. Additionally, seawater contains a spectrum of minerals and as such may avoid the addition of essential nutrients currently required for commercial fermentation media^9^. Thus, using seawater in fermentations could potentially improve the overall economics of the process and make a positive impact on overcoming both the freshwater and energy crises^7,10^. Some early investigations have been carried out to replace freshwater with seawater in bioenergy plant cultivation^11^, lignocellulosic raw material pre-treatment^12,13^, enzymatic hydrolysis^14^ and bioethanol fermentation stages of the production system^15^. However, bioethanol fermentation efficiency was low in the presence of seawater with a bioethanol concentration of no more than 11.60 g/L^15^. This is probably due to the high osmotic and inhibitor stresses that suppressed the fermentation capacities of the yeast strains used in these studies.

Current industrial yeast strains are predominately isolated from terrestrial environments. Utilising these yeast strains for bioethanol production using seawater-based media could be challenging as seawater has a high salt content (around 3.50%). Hence, in the last two decades, there have been continuous efforts to isolate and exploit marine microorganisms for bioethanol production and beyond^7,16^. Research on yeast isolated from marine environments revealed that these organisms have several promising features over terrestrial yeast strains, especially with regard to osmotic tolerance, cold adaptability and metabolism of marine derived simple sugars^7,17^. Marine yeast fermentation using freshwater and commercial glucose has resulted in bioethanol production of 122.50 g/L from 297 g/L of glucose^18^. In a fermentation of a red seaweed *Kappaphycus alvarezii* hydrolysate containing 5.50% reducing sugar, around 12.30 g/L bioethanol was obtained using a *Candida* sp. isolated from a marine environment^19^.

In this study, we report a detailed investigation on bioethanol production using seawater-based media and a new marine yeast strain ‘*S. cerevisiae* AZ65′. Various marine yeast strains were screened for bioethanol production. Then selected yeast strains were explored for their osmotic tolerance (salts and sugars) in comparison to an industrial terrestrial yeast *S. cerevisiae* NCYC2592. Finally, the fermentation efficiency of marine yeast using a medium prepared with seawater replacing freshwater was demonstrated in 15 L fermenters. Although the water requirements during the fermentation process was estimated at around 5 to 11 litres of water for producing one litre of bioethanol, the replacement of this amount of water with seawater will immensely increase the efficiency of the process at the industrial scale^20^.

## Materials and Methods

### Seawater preparation

Seawater (SW) was used in this paper for media preparation and dilutions. Seawater was obtained from Skegness, Lincolnshire, UK, which is located on the North Sea. The seawater was filtered using glass microfiber filters (pore size, 1.20 μm; Whatman^®^) and autoclaved at 121 °C for 15 min, then stored at 4 °C until required. Synthetic seawater (SSW) was prepared according to the formula suggested by Fang *et al*.^21^ and the compositions for 1X SSW and 2X SSW are shown in Supplementary Table ST1.

### Microorganisms

Nine marine yeast strains including; *Candida viswanathii* AZ8, *S. cerevisiae* AZ65, *S. cerevisiae* AZ71, *Wickerhamomyces anomalus* AZ80, *Pichia kudriavzevii* AZ83, *Issatchenkia orientalis* AZ88, *S. cerevisiae* AZ118, *Candida glabrata* AZ127 and *Candida albicans* AZ142 were used in this study. These strains were isolated, identified and characterized by Zaky *et al*.^22^. Propagation of each strain -from frozen stocks- was carried out using seawater-YPD (SW-YPD) medium containing (w/v) 1% yeast extract, 2% peptone, and 2% glucose at pH 6 ± 0.20 (seawater was used instead of freshwater for medium preparation). The propagation was performed aerobically in an orbital shaker (150 rpm) at 30 °C for 48 h. Working stock culture of each strain was prepared from the propagated cultures on SW-YPD agar slope (as above +2% agar) and kept at 4 °C.

*S. cerevisiae* NCYC2592 strain (www.ncyc.co.uk) was used in this study as a terrestrial reference yeast strain. It was maintained using the same method as the marine yeasts, but the YPD media were prepared using reverse osmosis water (ROW) instead of seawater. *S. cerevisiae* NCYC2592 is a distiller strain that shows high fermentation performance, and has been well characterised previously for sugar utilisation and tolerance to various inhibitory compounds. Hence, it was chosen in this study for the comparison with our new marine yeast strains^23,24^.

### Fermentation using Mini Fermentation Vessels (MFVs)

#### Preparation of MFVS

Fermentation experiments were conducted at a miniature scale (100 mL) using mini fermentation vessels (MFVs). The MFVs were assembled manually in the lab from the following items; i) 150 mL glass serum bottle (Wheaton, USA) ii) magnetic flea, iii) Bunsen valve, iv) rubber septum, vi) metal crimp. A magnetic flea was placed into the bottle which was then plugged with a cotton or sponge plug for sterilisation. Bottles, Bunsen valves and the rubber septa were sterilised separately at 121 °C for 15 min. Sterilised bottles were supplied with fermentation medium and the yeast inoculum then plugged with a rubber septum and sealed by a metal crimp to form anaerobic condition for fermentation. A Bunsen valve was inserted on the top to allow gas outlet (schematic diagram of MFV is presented in Supplementary Fig. SF1).

#### Inoculum preparation

Yeast inoculum was prepared as follows: i) a loopful of yeast growth, from the yeast slope, was inoculated into 20 mL of YPD broth in a 50-mL conical flask then incubated in an orbital shaker at 30 °C and 150 rpm for 48 h, ii) the culture was then transferred into a 500-mL conical flask containing 200 mL of YPD (2% glucose, 2% peptone, 1% yeast extract (w/v)) and incubated for another 48 h under the same conditions, iii) yeast cells were harvested using a benchtop centrifuge at 3000 rpm for 3 min. (Eppendorf, UK). The yeast pellets were then washed three times by dissolving and harvesting them with sterile ROW. Clean yeast pellets were re-suspended in sterile ROW to form a concentrated liquid yeast inoculum with an OD_600_ of 500. MFVs were inoculated with 100 µL of the concentrated yeast inoculum to reach a starting cell concentration of 0.50 at OD_600_. Depending on the experiment, SW or SSW was used to replace ROW for media preparation and inoculum preparation.

#### Fermentation media

Depending on the objective of each experiment, different fermentation media were used as follows:

- Screening for high ethanol producing marine yeast was conducted using YPD media prepared in SW (SW-YPD) and ROW (ROW-YPD) which contains 5.50% glucose, 2% peptone, 1% yeast extract (w/v) dissolved in either ROW or SW.
- Salt tolerance experiment was conducted using 7 different media as follows: i) ROW-YPD medium containing 5.50% glucose, 2% peptone, 1% yeast extract (w/v) dissolved in ROW, ii) NaCl-YPD medium containing 5.50% glucose, 2% peptone, 1% yeast extract (w/v) with the addition of 3%, 6% and 9% (w/v) NaCl dissolved in ROW, iii) SW-YPD medium containing 5.50% glucose, 2% peptone, 1% yeast extract (w/v) dissolved in SW, iv) SSW-YPD medium containing 5.50% glucose, 2% peptone, 1% yeast extract (w/v) dissolved in SSW and 2X SSW.
- Glucose tolerance experiment was conducted using SW based medium, containing 2% peptone, 1% yeast extract (w/v) and different concentrations of glucose (10, 15, 20, and 25% (w/v)).

All fermentation media were autoclaved at 121 °C for 15 min after adjusting pH to 6 using 1 N HCl and/or NaOH.

#### Culture conditions and data processing

Inoculated MFVs were then placed on a 15-position magnetic stirring plate (2mag, magnetic motion, Munich, Germany) set at 400 rpm and incubated in a static incubator at 30 °C for the required fermentation time. The rates of fermentation were monitored by measuring the weight loss (WL) using an analytical balance at regular time points during the fermentation until no further WL was observed. WL is analogous to the released CO_2_ from the Bunsen valve of the MFVs during fermentation. At the end of the fermentation, samples from each MFV were prepared for HPLC analysis to quantify the concentrations of glucose, ethanol and other metabolites, such as glycerol and acetic acid. All fermentations have been carried out in triplicate.

The Specific Fermentation Rate (SFR) was calculated for the exponential weight loss (WL) period using Equation 1. The maximum SFR was used to compare the fermentation rates of marine yeasts with the reference terrestrial yeast.

1 $$SFR=\frac{WL\,at\,sample\,point\,(n+1)-WL\,at\,sample\,point\,(n)}{WL\,at\,sample\,point\,(n)}$$

#### Fermentation using 15 L bioreactors

Large scale fermentations were conducted in a batch mode using 15 L, *in-situ* sterilisable, stainless steel bioreactors (Techfors-S, Infors-HT, Bottmingen, Switzerland) with 10 L working volumes.

#### Inoculum preparation

Inoculum of the marine yeast *S. cerevisiae* AZ65 were prepared for 10 L fermentation media using the following protocol: i) a loopful of yeast growth from the yeast slope was inoculated into 20 mL of SW-YPD medium in a 50-mL conical flask then incubated in an orbital shaker at 30 °C and 150 rpm for 48 h, ii) the culture was then transferred into a 500-mL conical flask containing 200 mL of SW-YPD medium and incubated for another 48 h under the same conditions, iii) 100 mL of the yeast culture was used to inoculate 1 L of SW-YPD medium in a 2 L conical flask and incubated at 30 °C and 150 rpm for 48 h, iv) yeast cells were harvested by centrifugation (Beckman, Model-J2-21) at 5000 rpm and 10 °C for 5 min., v) harvested yeast was washed three times by suspending and re-harvesting them with sterile SW, vi) clean yeast pellets were re-suspended in sterilised SW to form a concentrated slurry yeast inoculum with OD_600_ of 500. All steps were conducted under aseptic conditions.

#### Batch fermentation using marine yeast and YPD prepared using seawater (SW-YPD medium)

Fermenters were filled with 10 L of SW-YPD fermentation media containing 1% yeast extract, 2% peptone, and 20% glucose dissolved in seawater at a starting pH of 6. Sterilisation for the media and the bioreactor was conducted at 121 °C for 15 min. After autoclaving, fermentation medium was aerated for 1 h using compressed air at a rate of 10 L/min. Aerated media was aseptically inoculated with the concentrated yeast inoculum to achieve a final cell density of about 0.50 OD_600_. Fermentation was carried out at 30 °C and a stirring rate of 200 rpm. Samples were collected at regular time points. In order to achieve anaerobic condition during ethanol fermentation, no additional air or oxygen was injected into the vessels after the initial oxygen was consumed by the yeast. The fermentations were carried out in triplicate.

#### Batch fermentation using marine yeast and molasses prepared using seawater (SW-Molasses medium)

The molasses used in this study was a commercial product of sugarcane molasses (horse feed supplement grade) called ‘NAF Molasses’ that was purchased online from Amazon.co.uk. The total sugar content in this product was stated to be 45% (w/w). In order to remove unwanted inorganic particles, the crude molasses was treated as follows: 500 g of molasses was transferred into a measuring cylinder (1000 mL) and made up to 1 L with seawater to achieve a final concentration of 50% molasses (w/v). The diluted molasses was then transferred into a 2 L Duran bottle and supplemented by 5–10 mL of concentrated H_2_SO_4_ (98%) to lower the pH to 3.50, then 2 mL of 50% sterilised antifoam A (Sigma Aldrich, UK) was added. The bottle was then placed in an autoclave and heated at 100 °C for 45 min. The heated bottle was left to cool down to 55 °C then transferred into a cold room (4 °C) and left to stand overnight. Under aseptic conditions, the clear solution on the top of the molasses (about 70% of the total volume) was transferred into a sterile Duran bottle and the sediment was discarded.

For fermentation, the 15 L fermenter was firstly filled with 4 L of seawater then sterilized at 121 °C for 15 min. 6 L of the treated molasses solution was transferred aseptically into the bioreactor to obtain a molasses medium concentration of 30% (w/v). The medium was supplemented with 3 mL of antifoam (50% v/v, in seawater) and 10 mL of urea solution (20% w/v, in seawater) and the pH was adjusted to 5.50 using NaOH (50% w/v). The medium was aerated for 1 h using compressed air at a rate of 10 L/h. Aerated media was aseptically inoculated with the concentrated yeast inoculum to achieve a final cell density of about 1.0 OD_600_. Fermentation was carried out at 30 °C and stirring rate of 200 rpm. Samples were collected at regular time points. Anaerobic condition was achieved in the bioreactor as no air or oxygen was injected into the vessels during the fermentation. The fermentations were carried out in triplicate.

### HPLC analysis

Glucose, sucrose, fructose, glycerol, acetic acid and ethanol were analysed using a HPLC method developed by Zaky *et al*.^25^. Briefly, a JASCO HPLC system was used, consisting of a JASCO AS-2055 Intelligent auto sampler, a JASCO PU-1580 Intelligent pump (JASCO), and a JASCO RI-2031 Intelligent refractive index detector. The separation column was Hi-Plex H column (7.7 × 300 mm, 8 μm), operating at 35 °C. The mobile phase was 0.005 N H_2_SO_4_ at a flow rate of 0.40 mL/min. The injection volume was 10 μL and the total running time was 32 min^25^.

### Statistical analysis

Microsoft Excel was used to calculate data means and standard deviations. Statistical analysis was performed using the analysis of variation (ANOVA) with the statistical software GraphPad Prism6, (GraphPad, Software, Inc., CA, USA), a P-value < 0.05 was considered statistically significant.

## Results

### Screening for ethanol producing marine yeast

Using a small scale fermentations (100 mL), 9 marine yeasts were screened for ethanol production from YPD media containing 5.50% (w/v) glucose which had been prepared using ROW and SW. *S. cerevisiae* NCYC2592, an industrial distillers yeast was used as a terrestrial reference for comparison. The fermentation was carried out anaerobically at 30 °C for 30 h. The fermentation rate was monitored as a weight loss over the fermentation period. Glucose utilisation and fermentation output (ethanol, glycerol and acetic acid) were determined using HPLC.

**Figure 1:**
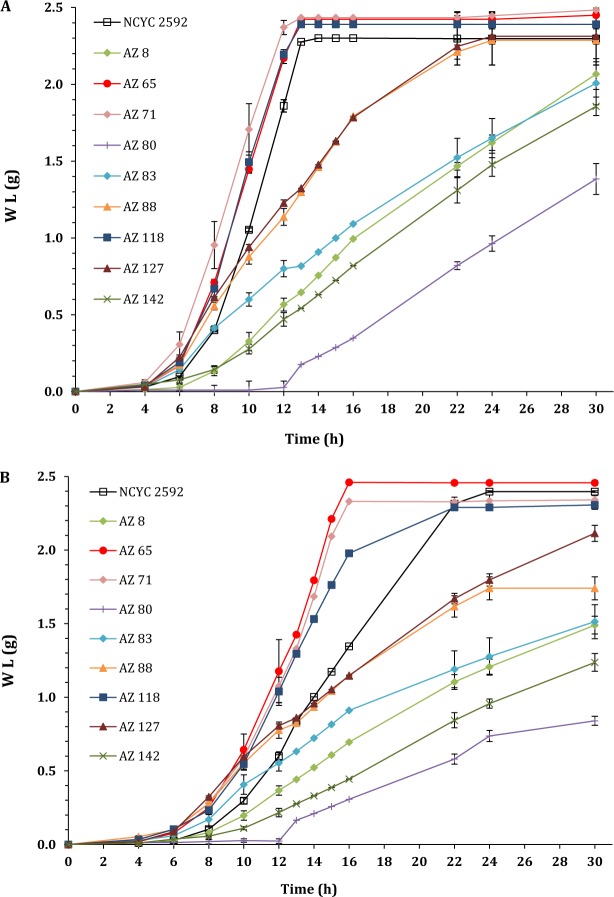
Screening for ethanol producing marine yeast using (**A**) SW-YPD medium and (**B**) ROW-YPD medium. AZ8 to AZ142 are nine marine yeast strains under investigation for bioethanol production NCYC2592 is a terrestrial *S*. *cerevisiae* strain used as a reference for comparison. Weight loss - as a result of CO_2_ release from the MFVs - was measured during the fermentation at different time intervals to assess the fermentation performance of each yeast strain. The experimental error bars represent the standard deviation of the three replicates.

In fermentations using ROW-YPD, marine *S. cerevisiae* strains (AZ65, AZ71, and AZ118) had faster fermentation rates when compared with the reference strain (*S. cerevisiae* NCYC2592). In general, non*-S. cerevisiae* yeasts had slower fermentation rates, however, two of them (*I. orientalis* AZ88 and *C. glabrata* AZ127) had faster fermentation rates in the first 8 h of the fermentation when compared with the reference strain (Fig. 1A). Fermentations using SW-YPD indicated that marine *S. cerevisiae* strains (AZ65 and AZ118) had significantly faster fermentation rates when compared with the reference strain (p < 0.05). Two non-*S. cerevisiae* marine strains (AZ88 and AZ127) had a fast rate of fermentation initially; but after 12 h, their fermentation rates slowed in comparison with the reference strain (Fig. 1B).

Tables 1 and 2 show glucose consumption, ethanol concentration, productivity and yield in these fermentations using ROW and SW based media. All *S. cerevisiae* strains - including the reference strain - utilised all of the glucose provided in the fermentation media (55 g/L) within 30 h of fermentation regardless of the type of water being used (ROW or SW) for media preparation. Moreover, two of the non-*S. cerevisiae* marine strains (*I. orientalis* AZ88 and *C. glabrata* AZ127) also utilised all the glucose present in the fermentation media but only when ROW was used for media preparation. The other non-*S. cerevisiae* strains did not fully consume the glucose provided in the media with either ROW or SW. Generally, glucose utilisation was faster when ROW was used in the fermentation media for all strains.

**Table 1:** HPLC analysis of fermentation samples of marine yeasts under screening for ethanol production using ROW-YPD medium.

ID Name	Strain No.	Time (h)	Consumed Glucose (%)	Glycerol (g/L)	EtOH (g/L)	EtOH Yield (%)^a^	EtOH Yield (%)^b^	EtOH Prod. (g/L/h)
*S. cerevisiae*	NCYC2592	14	100.00	1.92 ± 0.05	23.10 ± 0.25	82.36	82.36	1.65
*C. viswanathii*	AZ8	30	87.73	1.54 ± 0.09	19.99 ± 1.71	71.26	81.22	0.67
*S. cerevisiae*	AZ65	13	100.00	2.20 ± 0.19	22.98 ± 0.47	81.93	81.93	1.77
*S. cerevisiae*	AZ71	13	100.00	2.13 ± 0.16	22.86 ± 0.78	81.50	81.50	1.76
*W. anomalus*	AZ80	30	57.90	1.40 ± 0.01	12.99 ± 0.37	46.31	79.98	0.43
*P. kudriavzevii*	AZ83	30	81.28	1.92 ± 0.13	18.71 ± 0.80	66.69	82.05	0.62
*I. orientalis*	AZ88	30	100.00	3.67 ± 0.14	21.44 ± 0.37	76.44	76.44	0.71
*S. cerevisiae*	AZ118	13	100.00	1.89 ± 0.14	22.39 ± 0.14	79.82	79.82	1.72
*C. glabrata*	AZ127	30	100.00	3.73 ± 0.10	20.54 ± 0.33	73.24	73.24	0.68
*C. albicans*	AZ142	30	76.82	1.14 ± 0.03	17.18 ± 0.21	61.24	79.71	0.57

^a^Calculated as a percentage of the theoretical yield (0.51) based on the total glucose (55 g).

^b^Calculated as a percentage of the theoretical yield (0.51) based on the utilised glucose.

**Table 2:** HPLC analysis of fermentation samples of marine yeasts under screening for ethanol producing yeast using SW-YPD medium.

ID Name	Strain No.	Time (h)	Consumed Glucose (%)	Glycerol (g/L)	EtOH (g/L)	EtOHYield^a^ (%)	EtOH Yield^b^ (%)	EtOH Prod. (g/L/h)
*S. cerevisiae*	NCYC2592	24	100.00	2.38 ± 0.10	25.75 ± 0.58	91.80	91.80	1.07
*C. viswanathii*	AZ8	30	59.91	1.46 ± 0.05	16.14 ± 0.21	57.54	96.05	0.54
*S. cerevisiae*	AZ65	16	100.00	2.54 ± 0.04	25.94 ± 0.52	92.48	92.48	1.62
*S. cerevisiae*	AZ71	16	100.00	3.03 ± 0.08	23.59 ± 0.29	84.09	84.09	1.47
*W. anomalus*	AZ80	30	58.96	2.05 ± 0.18	13.83 ± 0.34	49.31	83.65	0.46
*P. kudriavzevii*	AZ83	30	74.87	2.29 ± 0.20	17.78 ± 1.90	63.39	84.66	0.59
*I. orientalis*	AZ88	30	76.25	3.33 ± 0.20	19.17 ± 0.81	68.34	89.62	0.64
*S. cerevisiae*	AZ118	22	100.00	2.93 ± 0.22	23.72 ± 1.16	84.58	84.58	1.08
*C. glabrata*	AZ127	30	80.31	4.00 ± 0.17	21.42 ± 0.77	76.38	95.11	0.71
*C. albicans*	AZ142	30	51.58	1.25 ± 0.11	13.99 ± 0.49	49.87	96.67	0.47

^a^Calculated as a percentage of the theoretical yield (0.51) based on the total glucose (55 g).

^b^Calculated as a percentage of the theoretical yield (0.51) based on the utilised glucose.

In fermentations using ROW, ethanol productivity of marine *S. cerevisiae* strains AZ65 and AZ71 were statistically higher than that of *S. cerevisiae* NCYC2592 (p = 0.04 and 0.03, respectively). When SW was used, ethanol productivity of these strains, AZ65 and AZ71, were 1.62 and 1.47 g/L/h, respectively, which was 51% and 38% higher than the reference strain (*S. cerevisiae* NCYC2592). Interestingly, ethanol yields (based on the utilised glucose) were always higher when SW-based media was used with all strains used in this study. This may be due to the fact that the presence of salt in the fermentation media decreases the growth of yeast cells^26,27^ so, less glucose was consumed for biomass production and, therefore, more glucose was converted to ethanol. The highest ethanol yield using ROW was 82.36% (calculated as a percentage of the theoretical yield), which was achieved by the reference strain while the highest ethanol yield using SW was 92.48%, which was achieved by the marine yeast *S. cerevisiae* AZ65.

Ethanol production ranged from 12.99 ± 0.37 to 23.10 ± 0.25 g/L using ROW-YPD and from 13.83 ± 0.34 to 25.94 ± 0.52 g/L using SW-YPD. The production of ethanol by 6 strains, including the reference strain, was higher using SW-based medium; while, 4 strains (AZ8, AZ83, AZ88 and AZ142) produced more ethanol using ROW-based medium. The production of glycerol was slightly higher when SW was used compared with using ROW and ranged from 1.25 ± 0.11 to 4.00 ± 0.17 and from 1.14 ± 0.03 to 3.73 ± 0.10 g/L respectively; however, two marine strains (AZ8 and AZ88), produced slightly higher amounts of glycerol using ROW in comparison with SW.

### Investigating the effect of glucose and salt concentrations on the fermentation performance of two marine *S. cerevisiae* strains

Screening for marine yeast with high fermentation performance revealed that two marine *S. cerevisiae* strains (AZ65 and AZ118) could be potential candidates for bioethanol production using SW-based fermentation media. These strains and the reference strain (*S. cerevisiae* NCYC2592) were further investigated for their fermentation ability in the presence of different salt concentrations (3–9% NaCl, SSW and 2X SSW). They were also investigated for their fermentation ability in the presence of different glucose concentrations (10, 15, 20 & 25%).

### Fermentation performance of marine *S. cerevisiae* under high salt concentrations

**Figure 2:**
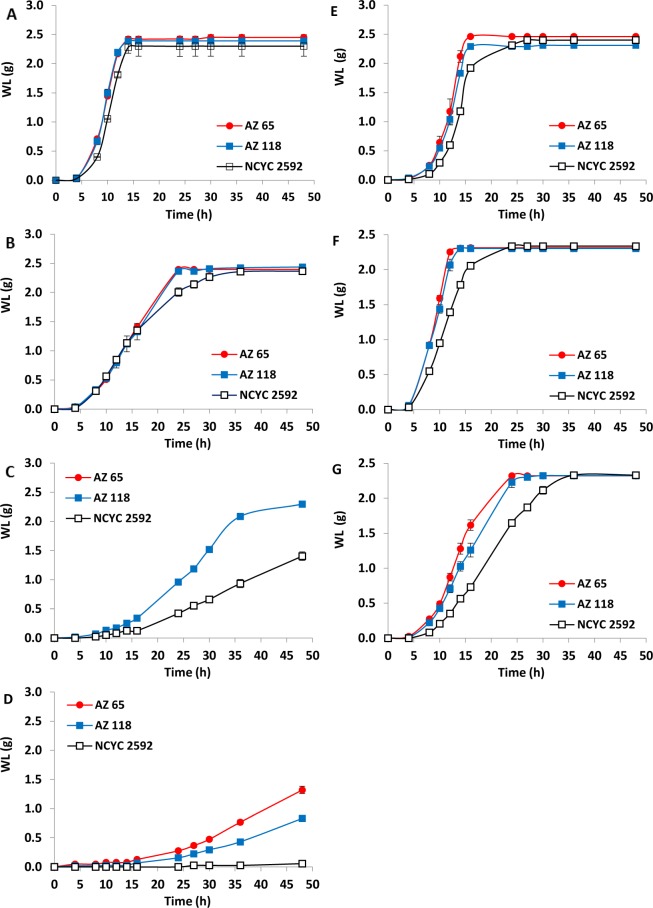
Fermentation performance of two marine yeast strains when exposed to salt stress using YPD media dissolved in different saline solutions (**A**) ROW, (**B**) NaCl 3%, (**C**) NaCl 6%, (**D**) NaCl 9%, (**E**) SW, (**F**) SSW, (**G**) 2X SSW. AZ65 & AZ118 are two marine *S. cerevisiae* strains. NCYC2592 is a terrestrial S. cerevisiae strain used as a reference for comparison. Weight loss as a result of CO_2_ release from MFVs was measured during the fermentation at different time intervals to assess the fermentation rate of each yeast strain. The experimental error bars represent the standard deviation of the three replicates.

When using ROW fermentation media, there were no differences in the fermentation rates for all strains in this study (Fig. 2A). Fermentations under different NaCl concentrations revealed that *S. cerevisiae* NCYC2592 had a slower rate of fermentation in media containing 3 and 6% of NaCl when compared with the marine strains (Fig. 2B,C). In addition, the results showed that fermentation media containing 9% NaCl inhibited the fermentation of *S. cerevisiae* NCYC2592; while fermentations with marine strains were characterised by a long lag phase before the fermentations commenced (Fig. 2D). Fermentations with marine yeast using SW-based medium were completed within 14 h, however, NCYC2592 required over 24 h before completion (Fig. 2E). Faster fermentation rates with shorter lag phases were obtained when SSW was used instead of SW (Fig. 2F). When 2X SSW fermentation medium was used, marine strains finished their fermentations within 24 h, however, *S. cerevisiae* NCYC2592 required 36 h before completion (Fig. 2G).

The Specific Fermentation Rate (SFR) for the three yeasts was calculated based on the WL data obtained under NaCl concentrations ranging from 0 to 9% which was reported in Fig. 2A–D, as shown in Supplementary Fig. SF2A. Data revealed that SFR of the reference strain (*S. cerevisiae* NCYC2592) dropped from 0.38 h^−1^ at 0% NaCl to 0.044 h^−1^ at 6% NaCl. The critical inhibitory NaCl concentration for *S. cerevisiae* NCYC2592 was estimated to be 6.40%. In contrast, the critical inhibitory NaCl concentration for *S. cerevisiae* AZ65 and AZ118 were 13.70% and 14.40%, respectively (Supplementary Fig. SF2A). Plotting the SFR of marine yeasts *S. cerevisiae* AZ65 and AZ118 in fermentations using SSW and 2X SSW into SF2B showed the data fit the trend lines very well. It was observed that *S. cerevisiae* NCYC2592 fermented faster in SSW and 2X SSW than those of in NaCl alone. This suggests that certain salt(s) in the SSW improved *S. cerevisiae* NCYC’s resistance to osmotic stress. This was also confirmed in the fermentations using natural SW. The SFR for all tested yeasts were clearly higher in fermentations using actual SW than that in fermentations with NaCl or SSW (Supplementary Fig. SF2B), suggesting that certain element(s) in seawater provide beneficial impacts for osmotic stress resistance.

### Fermentation performance of marine *S. cerevisiae* under high glucose concentrations

**Figure 3:**
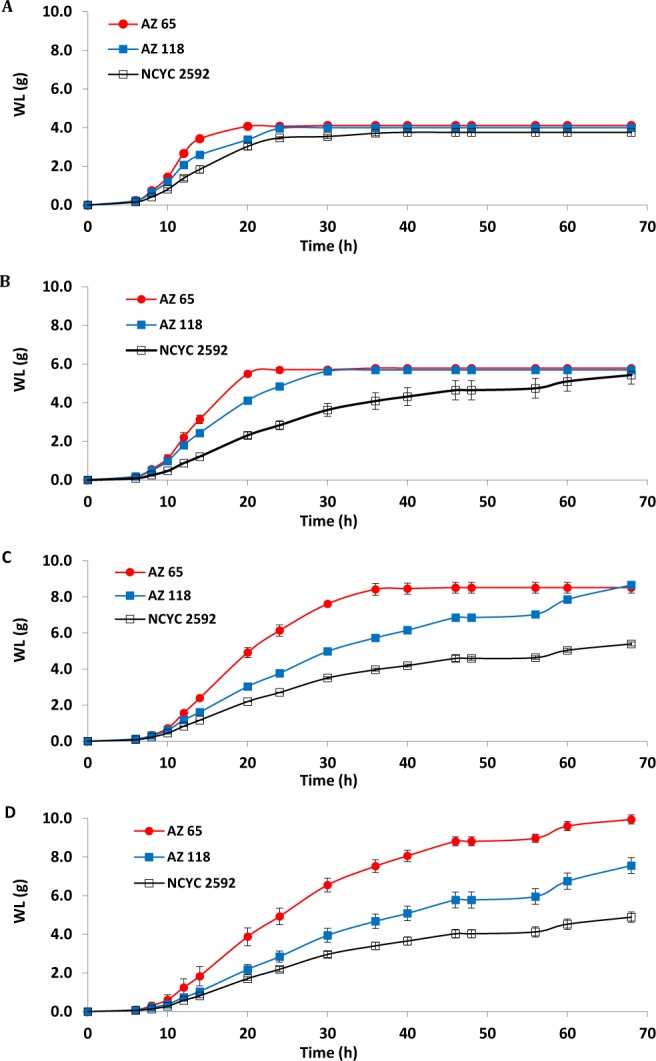
Fermentation performance of two marine yeast strains when exposed to osmotic stress using YPD media containing glucose concentrations (10, 15, 20, 25%) dissolved in seawater. (**A**) Glucose 10%, (**B**) glucose 15%, (**C**) glucose 20%, (**D**) glucose 25%. AZ65 & AZ118 are two maine *S. cerevisiae* strains. NCYC2592 is a terrestrial *S. cerevisiae* strain used as a reference for comparison. Weight loss as a result of CO_2_ release from MFVs was measured during the fermentation at different time intervals to assess the fermentation rate of each yeast strain. The experimental error bars represent the standard deviation of the three replicates.

Results revealed that marine strain *S. cerevisiae* AZ65 recorded the best fermentation rates with all glucose concentrations while the reference strain recorded the lowest fermentation rates (Fig. 3). Using media containing 10% glucose, all strains finished the fermentation within 68 h, however, reference strain NCYC2592 required 36 h, AZ118 required 24 h and AZ65 required 20 h to complete their fermentations (Fig. 3A). In case of media containing 15% glucose, all strains were also able to complete the fermentation, however, NCYC2595 required the maximum scheduled fermentation time of the experiment while AZ118 required 30 h and AZ65 required 24 h (Fig. 3B). When 20% glucose was added to the fermentation media, NCYC2592 did not complete the fermentation in the time-frame used for assessment and AZ118 required the maximum time of the experiment while AZ65 completed the fermentation within 36 h (Fig. 3C). No strain could complete the fermentation using SW-YPD media supplemented with 25% glucose. However, the weight loss, as a result of the ethanol fermentation, recorded by AZ65 was 9.95 g while AZ118 and NCYC2592 recorded 7.56 and 4.89 g, respectively (Fig. 3D). The results clearly indicated that the marine strain AZ65 was more tolerant to osmotic stresses compared to the reference and the other marine strains (AZ118), especially at a sugar concentration of 25%.

**Figure 4:**
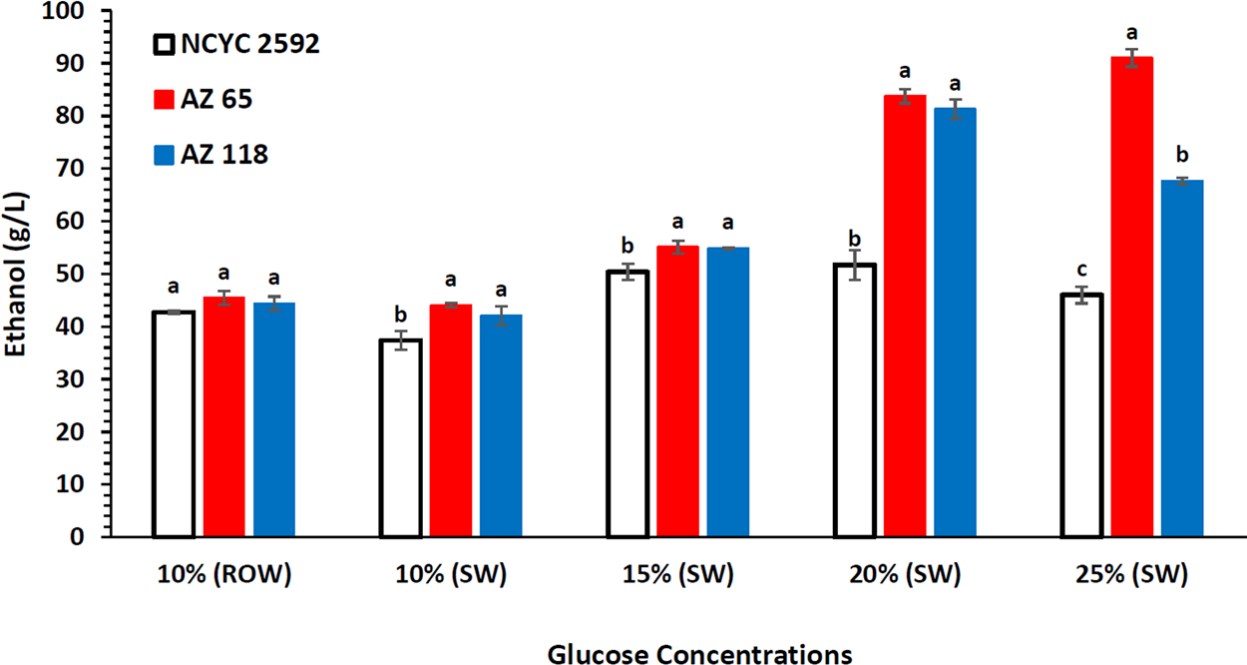
Comparing ethanol production by 2 marine strains and the reference strain using SW-YPD media containing increased glucose concentrations (10, 15, 20, 25%) dissolved in seawater. ^a,b,c^Columns bearing different superscript in the same treatment differ significantly (p < 0.05). The experimental error bars represent the standard deviation of the three replicates.

HPLC analyses for ethanol production revealed that the marine strain AZ65 produced significantly higher concentrations of ethanol when compared with the reference strain when using SW-YPD media at all glucose concentrations (Fig. 4). The results also revealed that AZ65 produced significantly higher concentrations of ethanol comparing with the other marine strain (AZ118) when using SW-YPD media containing 25% glucose. No significant difference in ethanol production between all yeast strains was observed when ROW-YPD medium containing 10% glucose was used (Fig. 4).

Table 3 detailed the fermentation output of these three strains using various glucose concentrations. The reference strain was able to utilise 100% of the glucose in the fermentation media during the experiment at the concentration of 10% only. The highest ethanol yield was 83.77% and highest ethanol productivity was 1.19 g/L/h both of which were obtained when ROW media containing 10% glucose was used. Ethanol yield and productivity generally decreased as the concentration of glucose in the SW media increased, the lowest yield and productivity were 36.05% and 0.68 g/L/h, respectively. The lowest glycerol and acetic acid were produced from ROW medium and recorded 4.92 ± 0.20 and 0.37 ± 0.01 g/L, respectively. The production of glycerol and acetic acid increased as the glucose was increased in the SW fermentation media and reached 10.50 ± 0.25 and 1.10 ± 0.04 g/L, respectively (Table 3).

**Table 3:** HPLC analysis for fermentations using SW-YPD media containing increased glucose concentrations (10–25%).

Strain	Medium Glucose (g/L)	Time (h)	Consumed Glucose (%)	Glycerol (g/L)	Acetic (g/L)	EtOH (g/L)	EtOH Yiel^a^ (%)	EtOH Yield^b^ (%)	EtOH Produc. (g/L/h)
NCYC2592	10% (ROW)	36	100.00	4.92 ± 0.20	0.37 ± 0.01	42.72 ± 0.27	83.77	83.77	1.19
	10%	40	100.00	7.60 ± 0.31	0.66 ± 0.01	37.36 ± 1.77	73.25	73.25	0.93
	15%	68	74.44	9.36 ± 0.49	0.74 ± 0.03	50.34 ± 1.55	65.80	88.40	0.74
	20%	68	57.22	10.00 ± 0.66	0.94 ± 0.06	51.73 ± 2.83	50.71	88.62	0.76
	25%	68	43.03	10.50 ± 0.25	1.10 ± 0.04	45.96 ± 1.57	36.05	83.77	0.68
AZ65	10% (ROW)	24	100.00	7.41 ± 1.25	0.37 ± 0.04	45.44 ± 1.31	89.10	89.10	1.89
	10%	30	100.00	7.91 ± 0.91	0.59 ± 0.02	44.07 ± 0.43	86.41	86.41	1.47
	15%	36	100.00	9.57 ± 0.35	0.64 ± 0.02	55.07 ± 1.25	71.98	71.98	1.53
	20%	46	100.00	12.57 ± 0.28	0.83 ± 0.03	83.75 ± 1.33	82.11	82.11	1.82
	25%	68	87.64	15.45 ± 0.84	1.08 ± 0.02	91.04 ± 1.70	71.40	81.47	1.34
AZ118	10% (ROW)	24	100.00	3.93 ± 0.34	0.16 ± 0.02	44.37 ± 1.31	87.00	87.00	1.85
	10%	30	100.00	6.78 ± 0.17	0.50 ± 0.04	42.10 ± 1.77	82.56	82.56	1.40
	15%	36	100.00	8.85 ± 0.42	0.70 ± 0.04	54.83 ± 0.17	71.67	71.67	1.52
	20%	46	91.99	12.58 ± 0.24	1.12 ± 0.04	81.28 ± 1.89	79.69	86.63	1.77
	25%	68	69.01	13.34 ± 0.89	1.36 ± 0.09	67.65 ± 0.64	53.06	76.89	0.99

^a^Calculated as a percentage of the theoretical yield (0.51) based on the total glucose used in the fermentation medium. ^b^Calculated as a percentage of the theoretical yield (0.51) based on the amount of utilised glucose by the end of the experiment.

The marine strain AZ118 recorded better results when compared with the reference strain. The strain utilised 100% of the available glucose in fermentation media that contained up to 15% glucose. The highest ethanol yield and productivity were obtained from ROW medium and recorded as 87% and 1.85 g/L/h, respectively. Ethanol yield and ethanol productivity in SW media ranged from 82.56 to 53.06% and 1.85 to 0.99 g/L/h, respectively. The lowest glycerol and acetic acid concentrations were produced from ROW medium and recorded 3.93 ± 0.34 and 0.16 ± 0.02 g/L, respectively. The production of glycerol and acetic acid increased as the concentrations of glucose increased in the SW fermentation media and reached 13.34 ± 0.89 and 1.36 ± 0.09 g/L, respectively (Table 3).

The best performance was obtained by the marine strain AZ65. This strain could utilise 87% of the glucose present in a fermentation medium that contained 25% glucose after 68 h of fermentation. In addition, this strain utilised 100% of the glucose present in all other fermentation media (10, 15, 20% glucose) in less than 50 h of fermentation. The highest ethanol yield and ethanol productivity were obtained from ROW medium and recorded 89.10% and 1.89 g/L/h, respectively. The lowest ethanol yield was 71.98% from SW with 15% glucose while the ethanol yield from the other SW media was above 80%. The best ethanol productivity from SW-based media was 1.82 g/L/h and was obtained using SW media of 20% glucose, while the lowest ethanol productivity was 1.34 g/L/h which was obtained when using SW media containing 25% glucose. The lowest glycerol and acetic acid were produced from ROW medium and recorded 7.41 ± 1.25 and 0.37 ± 0.01 g/L, respectively. The production of glycerol and acetic were increased as the glucose was increased in the fermentation media and reached 15.45 ± 0.84 and 1.08 ± 0.02 g/L, respectively (Table 3).

### Assessing bioethanol production of marine *S. cerevisiae* AZ65 using seawater-based media in 15 L bioreactors

#### Ethanol production by the marine yeast strain, S. cerevisiae AZ65, using SW-YPD medium

**Figure 5:**
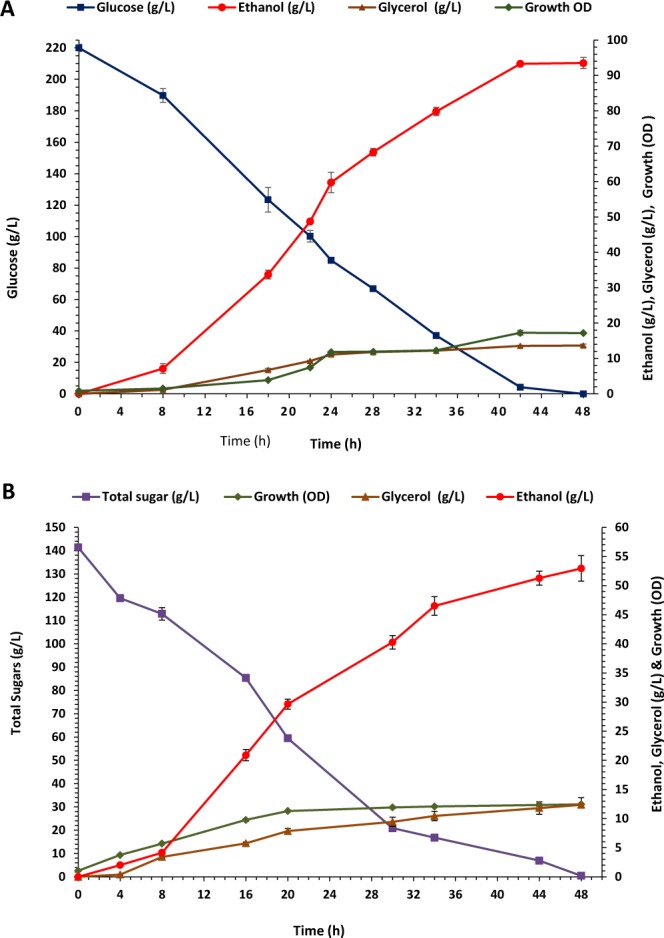
Changes in the concentration of glucose, ethanol, glycerol and biomass in fermentations conducted in 15 L bioreactor using SW-based medium and the marine strain *S. cerevisiae* AZ65. (**A**) SW-YPD medium; (**B**) SW-Molasses medium. The experimental error bars represent the standard deviation of the three replicates.

Fermentations revealed that the marine strain *S. cerevisiae* AZ65 was capable of efficiently converting high concentrations of glucose (20–25%) into ethanol using seawater-based media. Then, the performance of this strain in 15 L bioreactors was assessed. SW-YPD (20% glucose) fermentation medium was prepared using natural seawater and inoculated with the marine strain *S. cerevisiae* AZ65 at a rate of 0.86 ± 0.09 OD. Fermentation was conducted anaerobically at 30 °C and 200 rpm for 48 h. Yeast growth, ethanol concentration, glycerol concentration and the remaining glucose concentration were monitored at regular time intervals, and the results are shown in Fig. 5A and Table 4. Yeast growth reached a maximum OD of 17.25 ± 0.66 after 42 h. All available glucose was utilised by 48 h and there was a concurrent conversion of the glucose into ethanol. The maximum ethanol production (93.50 ± 1.59 g/L) was recorded at 48 h with 83.33% of the theoretical yield. Ethanol productivity increased during the first 24 h and reached 2.49 g/L/h, and then decreased during the second 24 h and reached 1.95 g/L/h by the end of fermentation. Glycerol production showed a consistent increase and reached a maximum concentration of 13.66 ± 0.43 g/L after 48 h of fermentation.

**Table 4:** HPLC analysis for fermentations conducted in 15-L bioreactors using SW-YPD media and the marine strain *S.cerevisiae* AZ65.

Time (h)	Growth (OD)	Consumed Glucose (%)	Glycerol (g/L)	EtOH (g/L)	EtOH Yield^a^ (%)	EtOH Yield^b^ (%)	EtOH Prod. (g/L/h)
0	0.86 ± 0.09	0.00	0.00 ± 0.01	0.00 ± 0.00	0.00	0.00	0.00
8	1.52 ± 0.03	13.75	1.15 ± 0.29	7.15 ± 1.39	6.38	46.16	0.89
18	3.88 ± 0.03	43.88	6.76 ± 0.48	33.72 ± 1.25	30.06	69.01	1.87
22	7.48 ± 0.03	54.45	9.30 ± 0.14	48.74 ± 0.19	43.44	79.79	2.22
24	11.84 ± 0.04	61.40	11.12 ± 0.41	59.75 ± 2.91	53.24	86.71	2.49
28	11.92 ± 0.01	69.58	11.78 ± 0.42	68.31 ± 1.06	60.89	87.49	2.44
34	12.31 ± 0.11	83.18	12.19 ± 0.16	79.81 ± 1.16	71.13	85.51	2.35
42	17.25 ± 0.66	98.06	13.57 ± 0.21	93.30 ± 0.36	83.16	84.80	2.22
48	17.18 ± 0.14	100.00	13.66 ± 0.43	93.50 ± 1.59	83.33	83.33	1.95

^a^Calculated as a percentage of the theoretical yield (0.51) based on the total glucose (220 g).

^b^Calculated as a percentage of the theoretical yield (0.51) based on the utilised glucose at the time of analysis.

#### Ethanol production by the marine yeast strain, S. cerevisiae AZ65, using SW-Molasses medium

The production of bioethanol using sugarcane molasses instead of commercial glucose was carried out. The fermentation was conducted under anaerobic conditions at 30 °C and 200 rpm for 48 h. The fermentation medium was prepared using sugarcane molasses at a concentration of 30% (w/v). The clarification and dilution for the molasses was done using seawater and the total initial sugars were determined by HPLC to be 138.8 ± 2.37 g/L. Yeast growth, ethanol concentration, glycerol concentration and the remaining sugar concentrations were monitored at regular time intervals (Fig. 5B and Table 5). Yeast growth reached a maximum OD of 12.44 ± 0.29 after 48 h of fermentation. Almost all available sugars (99.33%) were utilised by the end of the fermentation and there was a concurrent conversion of sugars into ethanol. It was noticed that the rate of sucrose utilisation was slower than that of glucose but faster than fructose. The final ethanol production was 52.23 ± 2.19 g/L with a yield of 73.80% of the theoretical yield. Ethanol productivity increased with time and reached a maximum of 1.43 g/L/h after 20 h then decreased to 1.09 g/L/h by the end of the fermentation. Glycerol production showed a consistent increase throughout the fermentation period and reached a maximum concentration of 13.17 ± 1.15 g/L.

**Table 5:** HPLC analysis for fermentations conducted in 15-L bioreactors using SW-Molasses media and the marine strain *S.cerevisiae* AZ65.

Time (h)	Growth OD	Sucrose (g/L)	Glucose (g/L)	Fructose (g/L)	Total Sugars (g/L)	Consumed Sugars (%)	Glycerol (g/L)	EtOH (g/L)	EtOH Yield^a^ (%)	EtOH Yield^b^ (%)	EtOH Prod. (g/L/h)
0	1.05 ± 0.05	18.03 ± 2.29	60.86 ± 0.40	59.92 ± 049	138.80 ± 2.37	0.00	0.00	0.00	0.00	0.00	0.00
4	3.74 ± 0.12	14.22 ± 2.41	53.30 ± 1.39	53.33 ± 2.42	120.84 ± 1.97	12.91	0.66 ± 0.27	1.88 ± 0.11	2.65	21.14	0.47
8	5.70 ± 0.21	13.27 ± 2.91	47.04 ± 2.75	49.65 ± 2.68	109.97 ± 6.54	20.79	2.74 ± 0.58	4.10 ± 0.51	5.79	28.94	0.51
16	9.76 ± 0.18	12.45 ± 3.06	29.80 ± 2.38	44.34 ± 2.09	86.60 ± 1.63	37.59	5.22 ± 0.65	18.36 ± 2.30	25.91	68.94	1.15
20	11.30 ± 0.17	10.13 ± 0.36	14.32 ± 1.97	35.53 ± 3.39	59.98 ± 5.38	56.79	7.88 ± 0.95	28.51 ± 2.90	40.25	71.36	1.43
30	11.91 ± 0.07	6.23 ± 0.25	3.33 ± 0.48	10.89 ± 2.57	20.45 ± 3.27	85.27	9.48 ± 0.40	41.87 ± 2.73	59.16	69.47	1.40
34	12.06 ± 0.10	5.53 ± 0.79	1.77 ± 1.54	6.90 ± 2.36	14.21 ± 4.68	89.79	10.67 ± 0.35	48.44 ± 2.86	68.48	76.21	1.42
44	12.34 ± 0.26	1.69 ± 0.28	0.75 ± 1.30	1.93 ± 1.65	4.37 ± 2.47	96.87	12.54 ± 1.19	51.76 ± 4.82	73.15	75.48	1.18
48	12.44 ± 0.29	0.87 ± 0.71	0.05 ± 0.09	0.00	0.92 ± 0.65	99.33	13.17 ± 1.15	52.23 ± 2.19	73.80	74.31	1.09

^a^Calculated as a percentage of the theoretical yield (0.51) based on the total sugar (138.8 g).

^b^Calculated as a percentage of the theoretical yield (0.51) based on the utilised glucose.

## Discussion

Coastal environments have been identified as being amongst the most diverse and microbially rich environments^28^. Although, marine fungi have been reported to have an active role in utilising available nutrients in marine environments^29^, their suitability for fermentations under osmotic stress conditions has not been investigated intensively. In this paper, the evaluation on the suitability of marine yeasts for ethanol production revealed that the marine strains, *S. cerevisiae* AZ65 and AZ118, performed significantly better than the reference strain (terrestrial *S. cerevisiae* NCYC2592) when seawater was used to prepare the fermentation media. It was observed that fermentation rates of all non-*S. cerevisiae* strains were slower than that of the reference strain, regardless of the type of fermentation media being used (Fig. 1). This finding supports *S. cerevisiae* as the preferred microorganism species for ethanol production.

Osmotic stress induced by the presence of salts is an important factor that affects yeast’s performance during fermentation^30^. Presence of salts of any kind has been shown to reduce glucose utilisation, cell growth and production of ethanol^27^. Improving salt tolerance has been highlighted as an important parameter for improving yeast performance in fermentation^26,31^. *S. cerevisiae* is a salt-sensitive yeast while other yeasts such as *Zygosaccharomyces rouxii* have been shown to be more tolerant to the presence of salt^32^. However, the sequencing of the *S. cerevisiae* S288C genome^33^ has led to insights into how *S. cerevisiae* responds to high external salt concentrations leading to conclusions on how the yeast modifies its genetic make-up to accommodate the changes in the extracellular environment^34^. In this study, results obtained from ethanol fermentation experiments revealed that marine *S. cerevisiae* AZ65 and AZ118 both had a higher tolerance to the presence of salts in the natural seawater, synthetic seawater and saline solutions containing up to 9% NaCl, when compared with the terrestrial reference yeast *S. cerevisiae* NCYC2592. This finding indicates that yeasts of marine origin have higher tolerance to osmotic stress induced by the presence of salts^35^. This observation supports a pervious finding that surrounding environment could play a significant role in improving the tolerance of *S. cerevisiae* to stresses induced by the presence of salts^34^.

It was also observed that fermentation rates using SSW-based media were higher than those using SW-based media even though both types of media contained a similar profile of salts. This suggested that natural seawater might contain unidentified inhibitors such as trace minerals. Studying these inhibitors may lead to way to avoid their effects (by water treatment or yeast modification) on yeast growth and the efficiency of fermentation. It was also observed that fermentation rates using a medium containing 3% NaCl was slower than fermentation rates using SSW-based medium (3.50% total salts) and similar to the fermentation rates obtained in 2X SSW-based medium (7% total salts). This could suggest that some salts, other than NaCl, may have a positive role in relieving the inhibitory effect of NaCl on yeast.

In fermentations using high concentrations of glucose in seawater, the marine *S. cerevisiae* AZ65 strain performed significantly better than the reference terrestrial strain (Fig. 4). Urano *et al*. (2001) studied the fermentation ability and salt tolerance of yeasts isolated from various aquatic environments (upper stream of Arakawa river, middle and lower streams of Tamagawa rivers and sea coasts of Kemigawa in Chiba prefecture and of Chemigahama in Choshi city) and concluded that yeasts with higher salt tolerance and higher fermentation ability were marine yeasts^35^. Khambhaty *et al*.^19^ validated the ability and efficiency of a marine isolate (*Candida* sp.) to grow and ferment galactose to ethanol in the presence of different types of salts (NaCl, CaCl_2_, and KCl) and at different concentrations (0–15%). This yeast strain yielded 1.23 to 1.76% ethanol from seaweed hydrolysates, containing different concentrations of sugar (2.70 to 5.50%) and salt (6.25 to 11.25%)^19^.

Low ethanol yield was obtained by the reference strain in fermentations using media with high sugar content (>15%). Liu *et al*.^36^ claimed that fermentations conducted in stressful environments involving very high gravity medium results in incomplete utilisation of glucose at the end of fermentation. In addition, the stressful condition leads to slow yeast growth and low cell viability that will lead to a lower ethanol production^36^. However, ethanol yields were very similar in seawater-based media containing up to 20% glucose using our marine yeast (*S. cerevisiae* AZ65) investigated in this study. This finding indicates that *S. cerevisiae* AZ65 is a promising candidate for bioethanol industry especially when the fermentation media contain high amount of salts as in case of cellulosic and seaweed ethanol.

Fermentations in 15-L bioreactors were applied using the new marine strain *S. cerevisiae* AZ65 to explore their performance in seawater fermentations at elevated scales. Using SW-YPD medium containing 22% glucose confirmed that *S. cerevisiae* AZ65 is an efficient yeast for industrial ethanol production. Zaky *et al*., (2014) recently reviewed bioethanol production using marine yeasts^7^. Various ethanol titres have been reported, ranging from 12.30 to 68.50 g/L with one exception of 122.50 g/L^7,18^. In comparison with these results, the ethanol concentration achieved by *S. cerevisiae* AZ65 was amongst the highest that reported in the literature. These findings indicate the commercial potential of *S. cerevisiae* AZ65, especially since the results in this study were obtained in fermentations using seawater rather than freshwater. Ethanol yield in this study could have been enhanced by supplying a low amount of air or oxygen during the fermentation (micro-aerobic condition). Liu *et al*., (2016) found that supplying low amounts of oxygen during a very high gravity ethanol fermentation enhanced the yield and productivity of ethanol^36^. Oxygen advances cell recovery through the TCA cycle and respiration pathway by retaining vital cellular components during synthesis and carbon utilisation. Oxygen helps yeast synthesise sterols and unsaturated fats required for maintaining a healthy cell membrane^37^.

In this study, elevated fermentation at 15-L scale was applied using an industrial substrate (molasses) prepared in seawater to further validate the suitability of a seawater fermentation strategy at the industrial level and to explore the potential of the marine *S. cerevisiae* AZ65 strain for use within the bioethanol industry. Although, sucrose is the major sugar in raw molasses, and accounts for about 50% of the total sugars, the chemical analysis of our clarified molasses showed lower amounts of sucrose when compared with glucose and fructose. This was probably due to the addition of concentrated sulphuric acid (H_2_SO_4_) and heating for 1 h during molasses preparation^38^. In line with results obtained by D’Amore *et al*.^39^, we noticed that yeast favours the utilising of glucose, then sucrose and lastly fructose when they are present together in the fermentation medium. These fermentations only used seawater and molasses and no additional minerals were added indicating that seawater has the capacity to provide the essential minerals for cell growth and ethanol production^39^.

Results obtained in the current study demonstrated the potential for the application of marine yeast and seawater in bioethanol production. Typical industrial scale bioreactors of corn ethanol production contain around 76% water, 12% ethanol and 12% of solids by the end of the fermentation. Hence, If seawater was used in the fermentation, roughly 7 litres of high quality freshwater (distilled) can be obtained with each litre of ethanol produced (Supplementary Fig. SF3). Further advantages of using seawater in fermentations for bioethanol production include; a) the minerals in seawater will potentially reduce the need to add minerals to the fermentation media, b) the production of sea salt as an additional product, and c) production of salted high protein animal feeds that can be used to eliminate the cost of adding minerals to the animal diets. In addition, the salt content in seawater is not favourable for terrestrial microorganisms and therefore may play a role in controlling microbial contamination in the bioreactors.

In addition to bioethanol production, seawater fermentation approach has been suggested for baker’s yeast production^40^ and succinic acid production^9^. The advancement of seawater fermentation approach could include the use of marine substrates, especially seaweed, as a substrate for fermentation accommodating the concept of marine fermentation where the whole system is run on marine elements (seawater, marine substrates, and marine microorganisms) (Supplementary Fig. SF4)^8,41^. Thus, using seawater in fermentations could potentially improve the overall economics of the fermentation process and have a strong impact on overcoming freshwater, food and energy crises.

Fermentations using seawater occurs in the same manner as fermentation using fresh water on lab scale. However, on an industrial scale, corrosion may be an issue when using metal pipes in the system. This issue could be avoided by replacing the metal pipes with Polyvinyl Chloride (PVC) and Chlorinated Polyvinyl Chloride (CPVC) pipes that are already widely used in various applications. Corrosion may also occur inside the fermenters, especially in an aerobic fermentation, but the addition of a coating material to existing fermenters or use of corrosion resistant steel for manufacturing new fermenters could solve this problem.

## Conclusion

The marine environment has a huge potential as a source for new yeast isolates with promising properties. The marine *S. cerevisiae* AZ65 strain showed excellent fermentation capability using SW-based media when compared with the reference terrestrial yeast *S. cerevisiae* NCYC2592 and the other marine yeasts belong to different species. Experiments under high osmotic stress induced by the presence of salt and/or glucose, revealed that marine *S. cerevisiae* AZ65 is a halotolerant and osmotolerant yeast strain. *S. cerevisiae* AZ65 tolerated up to 9% NaCl and converted glucose into ethanol efficiently in SW-based medium containing up to 25% glucose. Fermentations in 15 L bioreactors using *S. cerevisiae* AZ65 produced 93.50 and 52.23 g/L ethanol from SW-YPD medium and SW-Molasses medium, respectively. The results indicated that the marine yeast strain *S. cerevisiae* AZ65 is a promising ethanol producer under osmotic stress.

## Electronic supplementary material

Supplementary Dataset

## Data Availability

The datasets and yeast strains used in the current study are available from the corresponding author (ASZ) on reasonable requests.

## References

[1] EIA, U.S. Energy Information Administration. International Energy Outlook 2016 with Projections to 2040. DOE/EIA-0484(2016). Preprint at https://www.eia.gov/outlooks/ieo/pdf/0484(2016).pdf.

[2] Yeh AC, Bai H (1999). Comparison of ammonia and monoethanolamine solvents to reduce CO_2_ greenhouse gas emissions. Science of The Total Environment.

[3] REN21. (Renewables 2014 Global Status Report (Paris: REN21 Secretariat). ISBN 978-3-9815934-2-6. Preprint at http://www.ren21.net/Portals/0/documents/Resources/GSR/2014/GSR2014_full%20report_low%20res.pdf (2014).

[4] OECD/Food and Agriculture Organization of the United Nations (2016), “Biofuels”, in OECD-FAO Agricultural Outlook 2016-2025, OECD Publishing, Paris. 10.1787/agr_outlook-2016-13-en (2016).

[5] Gerbens-Leenes W, Hoekstra AY, van der Meer TH (2009). The water footprint of bioenergy. Proceedings of the National Academy of Sciences.

[6] Gerbens-Leenes W, Hoekstra AY (2012). The water footprint of sweeteners and bio-ethanol. Environment International.

[7] Zaky AS, Tucker GA, Daw ZY, Du C (2014). Marine yeast isolation and industrial application. FEMS Yeast Research.

[8] Greetham D, Zaky A, Makanjuola O, Du C (2018). A brief review on bioethanol production using marine biomass, marine microorganism and seawater. Current Opinion in Green and Sustainable Chemistry.

[9] Lin CSK, Luque R, Clark JH, Webb C, Du C (2011). A seawater-based biorefining strategy for fermentative production and chemical transformations of succinic acid. Energy & Environmental Science.

[10] Serra, I. *et al*. Seawater-based biocatalytic strategy: stereoselective reductions of ketones with marine yeasts. *Chem Cat Chem*, 10.1002/cctc.201600947 (2016).

[11] Yuan WJ, Zhao XQ, Ge XM, Bai FW (2008). Ethanol fermentation with *Kluyveromyces marxianus* from Jerusalem artichoke grown in salina and irrigated with a mixture of seawater and freshwater. Journal of Applied Microbiology.

[12] vom Stein T (2010). Salt-assisted organic-acid-catalyzed depolymerization of cellulose. Green Chemistry.

[13] Senthilraja P, Kathiresan K, Saravanakumar K (2011). Comparative analysis of bioethanol production by different strains of immobilized marine yeast. Journal of Yeast and Fungal Research.

[14] Grande PM, Domínguez de María P (2012). Enzymatic hydrolysis of microcrystalline cellulose in concentrated seawater. Bioresource Technology.

[15] Gonçalves FA, Santos ESd, de Macedo GR (2015). Alcoholic fermentation of *Saccharomyces cerevisiae*, *Pichia stipitis* and *Zymomonas mobilis* in the presence of inhibitory compounds and seawater. Journal of Basic Microbiology.

[16] Ramesh S, Rajesh M, Mathivanan N (2009). Characterization of a thermostable alkaline protease produced by marine *Streptomyces fungicidicus* MML1614. Bioprocess and Biosystems Engineering.

[17] Sarkar S, Pramanik A, Mitra A, Mukherjee J (2010). Bioprocessing data for the production of marine enzymes. Marine Drugs.

[18] Obara N, Ishida M, Hamada-Sato N, Urano (2012). N. Efficient bioethanol production from scrap paper shredder by a marine *Saccharomyces cerevisiae* derived C-19. Studies in Science and Technology.

[19] Khambhaty Y (2013). Bioethanol from macroalgal biomass: utilization of marine yeast for production of the same. BioEnergy Research.

[20] Shapouri, H., Gallagher, P. Ethanol cost-of-production survey. *U.S. Department of Agriculture*. Preprint at http://www.ethanolrfa.org/wp-content/uploads/2015/09/usdacostofproductionsurvey.pdf (2005)

[21] Fang C (2015). Seawater as alternative to freshwater in pretreatment of date palm residues for bioethanol production in coastal and/or arid areas. ChemSusChem.

[22] Zaky A, Greetham D, Louis E, Tucker G, Du C (2016). A New Isolation and Evaluation Method for Marine Derived Yeast spp with Potential Applications in Industrial Biotechnology. Journal of microbiology and biotechnology.

[23] Greetham D (2014). Development of a phenotypic assay for characterisation of ethanologenic yeast strain sensitivity to inhibitors released from lignocellulosic feedstocks. Journal of Industrial Microbiology & Biotechnology.

[24] Oshoma CE (2015). Screening of non- *Saccharomyces cerevisiae* strains for tolerance to formic acid in bioethanol fermentation. PLOS ONE.

[25] Zaky AS, Pensupa N, Andrade-Eiroa Á, Tucker GA, Du C (2017). A new HPLC method for simultaneously measuring chloride, sugars, organic acids and alcohols in food samples. Journal of Food Composition and Analysis.

[26] Wei CJ, Tanner RD, Malaney GW (1982). Effect of sodium chloride on bakers’ yeast growing in gelatin. Applied and Environmental Microbiology.

[27] Casey E (2013). Effect of salts on the co-fermentation of glucose and xylose by a genetically engineered strain of *Saccharomyces cerevisiae*. Biotechnology for Biofuels.

[28] Danovaro R, Fonda Umani S, Pusceddu A (2009). Climate change and the potential spreading of marine mucilage and microbial pathogens in the Mediterranean Sea. PLoS One.

[29] Gao L, Liu X (2010). Nutritional requirements of mycelial growth and sporulation of several biocontrol fungi in submerged and on solid culture. Microbiology.

[30] Casey E, Sedlak M, Ho NW, Mosier NS (2010). Effect of acetic acid and pH on the cofermentation of glucose and xylose to ethanol by a genetically engineered strain of *Saccharomyces cerevisiae*. FEMS Yeast Researsh.

[31] Ramos CL (2013). Evaluation of stress tolerance and fermentative behavior of indigenous *Saccharomyces cerevisiae*. Brazilian Journal of Microbiology.

[32] Dakal TC, Solieri L, Giudici P (2014). Adaptive response and tolerance to sugar and salt stress in the food yeast *Zygosaccharomyces rouxii*. International Journal of Food Microbiology.

[33] Goffeau A (1996). Life with 6000 genes. Science.

[34] de Nadal E, Ammerer G, Posas F (2011). Controlling gene expression in response to stress. Nature Reviews Genetics.

[35] Urano N, Yamazaki M, Ueno R (2001). Distribution of halotolerant and/or fermentative yeasts in aquatic environments. Journal of the Tokyo University of Fisheries.

[36] Liu C-G, Hao X-M, Lin Y-H, Bai F-W (2016). Redox potential driven aeration during very-high-gravity ethanol fermentation by using flocculating yeast. Scientific Reports.

[37] Fornairon-Bonnefond C, Demaretz V, Rosenfeld E, Salmon J-M (2002). Oxygen addition and sterol synthesis in *Saccharomyces cerevisiae* during enological fermentation. Journal of Bioscience and Bioengineering.

[38] Bower S, Wickramasinghe R, Nagle NJ, Schell DJ (2008). Modeling sucrose hydrolysis in dilute sulfuric acid solutions at pretreatment conditions for lignocellulosic biomass. Bioresource Technology.

[39] D’Amore T, Russell I, Stewart GG (1989). Sugar utilization by yeast during fermentation. Journal of Industrial Microbiology.

[40] Kyyaly, M. A., Zaky, A. S. & Powell, C. D. Production of Baker’s yeast using seawater-based media. *New Biotechnology* **33**, Supplement, S71, 10.1016/j.nbt.2016.06.963 (2016).

[41] Zaky AS (2017). Marine Fermentation, the Sustainable Approach for Bioethanol Production. EC Microbiology.

