# Supplementary material for: The establishment of a marine focused biorefinery for bioethanol production using seawater and a novel marine yeast strain
Source: Sci Rep. 2018 Aug 14;8:12127. doi: 10.1038/s41598-018-30660-x (PMC6092365; doi:10.1038/s41598-018-30660-x)
Supplement: Supplementary file 1 — Supplementary Dataset [file 41598_2018_30660_MOESM1_ESM.pdf]

Electronic Supplementary Information for:

The establishment of marine focused biorefinery for bioethanol production using seawater and a novel marine yeast strain

Abdelrahman Saleh Zaky* [a, b, c], Darren Greetham [a, b], Gregory A. Tucker [b] and Chenyu Du* [a, b]

[a] School of Applied Sciences, University of Huddersfield, Huddersfield HD1 3DH, UK.

[b] School of Biosciences, University of Nottingham, Nottingham LE12 5RD, UK.

[c] Department of Microbiology, Faculty of Agriculture, Cairo University, Giza 12613, Egypt.

*** Corresponding authors:**

Dr Abdelrahman Zaky: A.Zaky@cu.edu.eg

Dr Chenyu Du: C.Du@hud.ac.uk

Contents

Seven pages (including the cover sheet)

One Table (ST1), four figures (SF1 to SF4) and References

Table ST1. The composition of synthetic seawater used in this study.

	Concentration g/L	
**Component**	**Standard (1X SSW)**	**Double strength (2X SSW)**
**NaCl**	27.133	54.266
**$MgCl_2$**	2.504	5.008
**$MgSO_4$**	3.382	6.764
**$CaCl_2$**	1.167	2.334
**KCl**	0.742	1.484
**$NaHCO_3$**	0.207	0.414
**NaBr**	0.085	0.17
**Total salts**	**35.22**	**70.44**

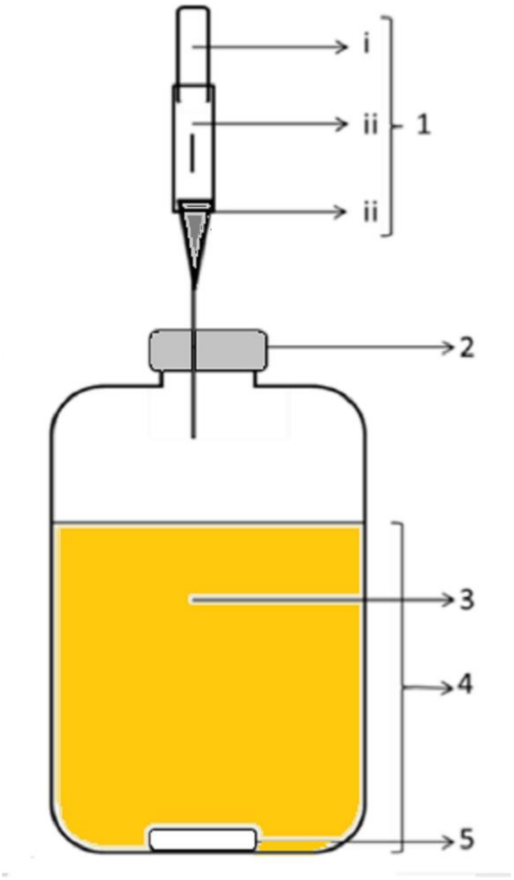

SF1: Schematic diagram of Mini Fermentation Vessel (MFV) for anaerobic fermentation

1, Bunsen valve (i, Durham tube; ii, Silicone tubing with a small cut in the middle; iii, Hypodermic needle), 2, rubber septum with metal crimp; 3, glass bottle; 4, working volume (100 mL); 5, magnetic flea.

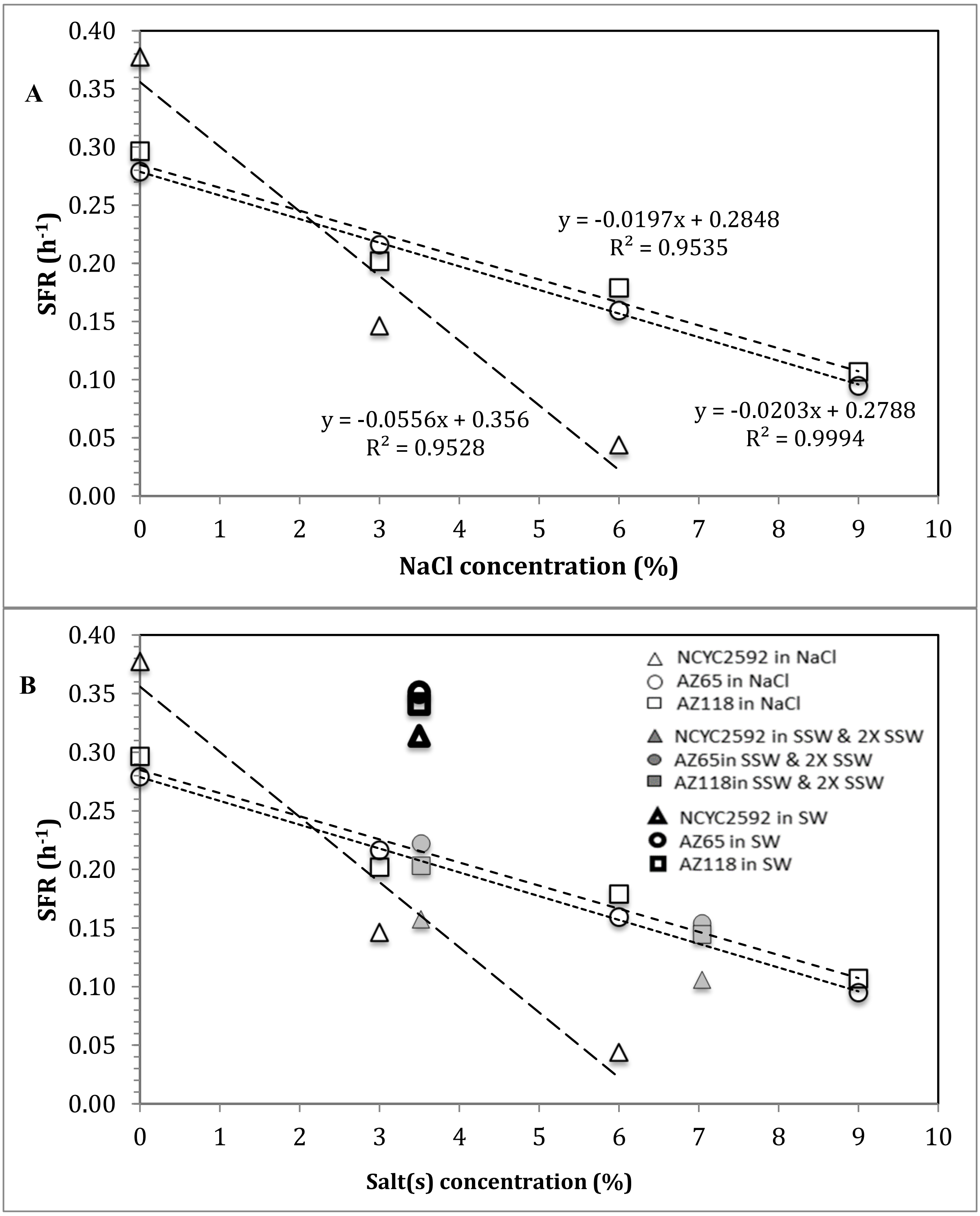

SF2: Specific Fermentation Rate (SFR) of marine yeasts (*S. cerevisiae* AZ65, AZ118) and the reference terrestrial yeast (*S. cerevisiae* NCYC 2592) in media containing different salt concentrations

(A) Calculating SFR of **yeasts** in media containing different NaCl concentrations (0-9%). The calculation was conducted based on the WL data presented in Figures 2A-2D.

(B) Comparing the SFR of yeasts in media containing different salt concentrations (NaCl (0-9%), SW, SSW & 2XSSW) . The calculation was conducted based on the WL data presented in Figures 2A-2G.

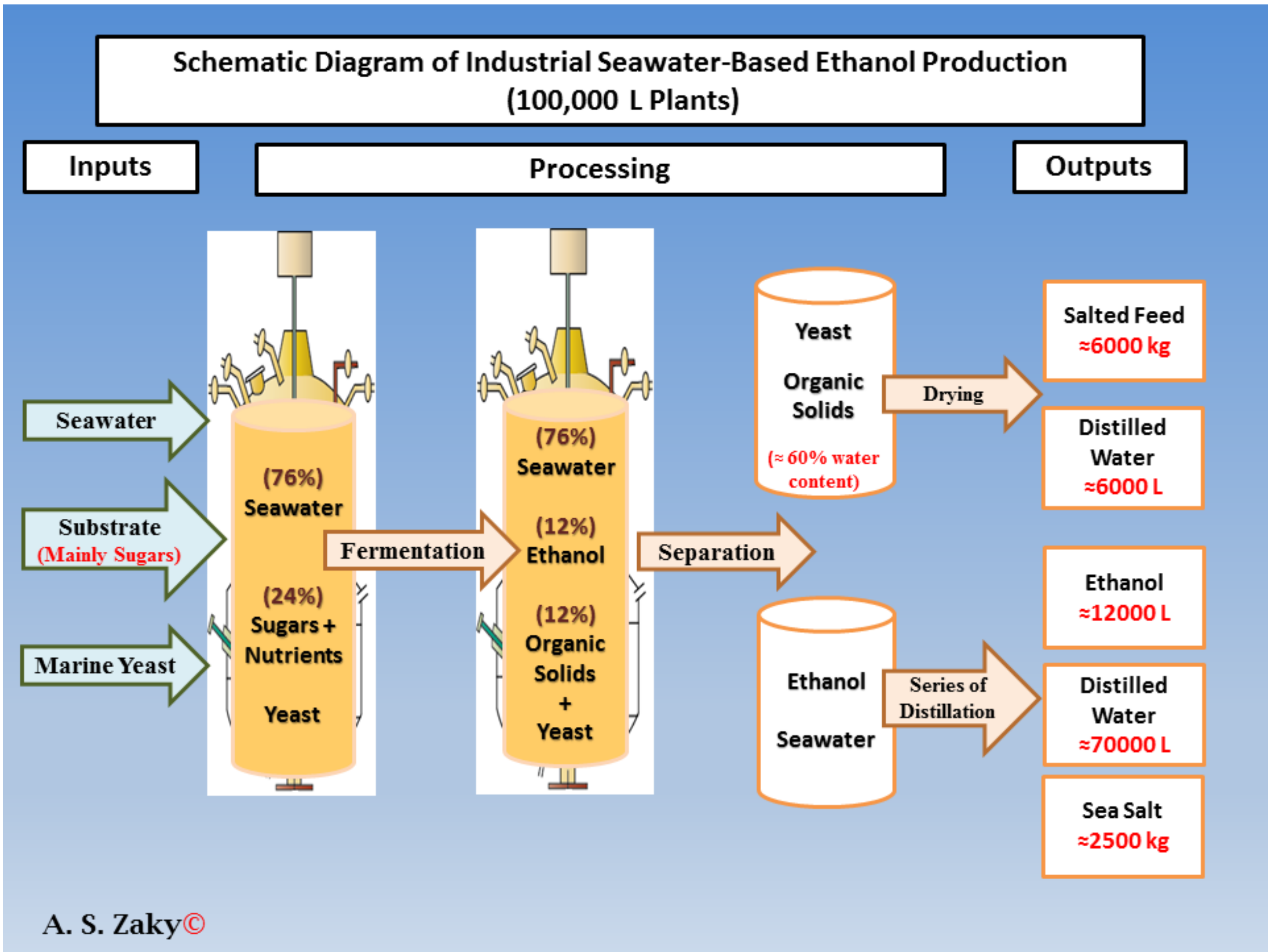

SF3: Schmatic diagram of industrial seawater-based ethanol production (100,000 L plants) The content of this diagrame was firstly suggested by Zaky in 2017 [1].

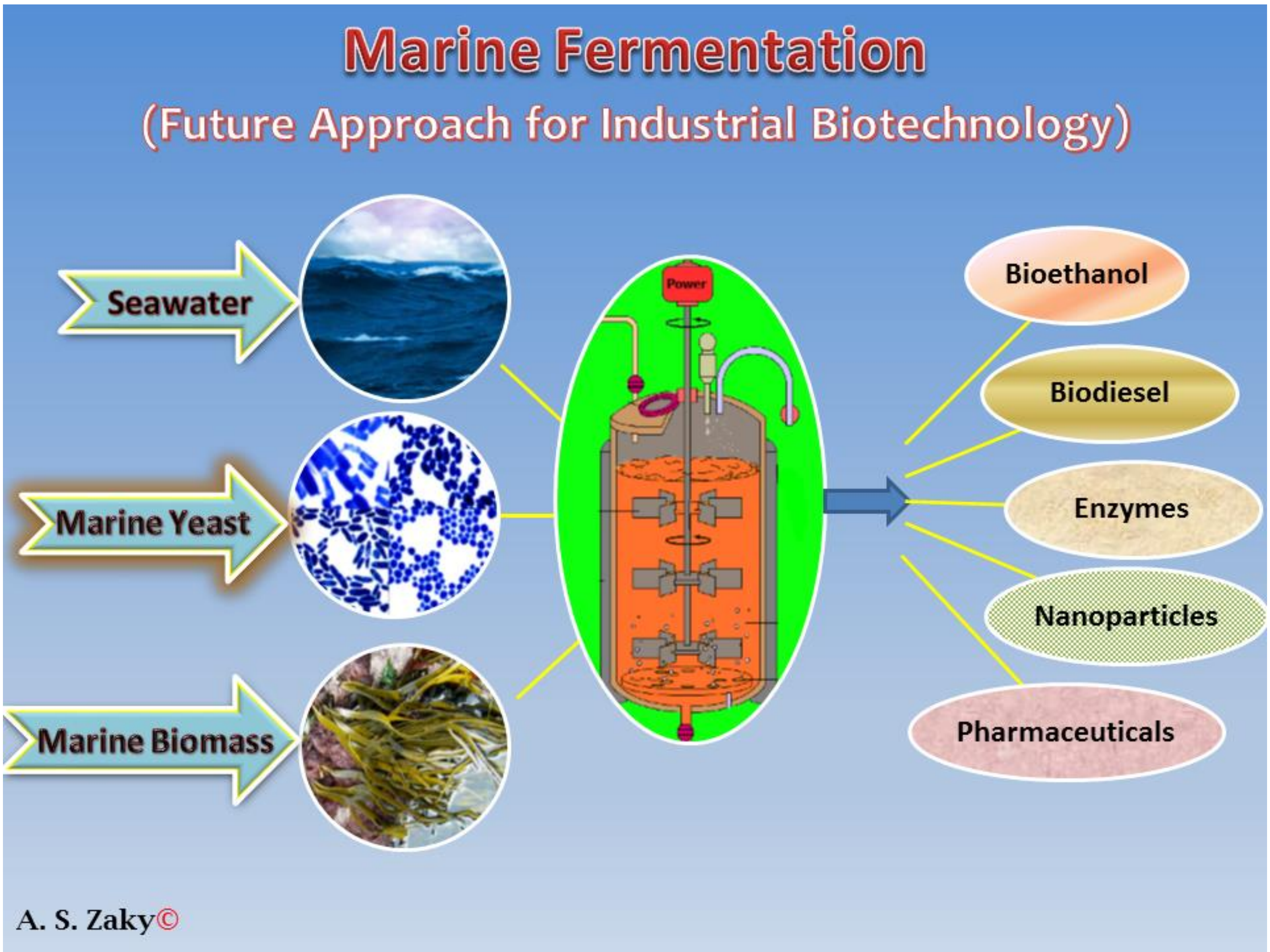

SF4: Schmatic diagram of the elements of Marine fermentation.
The concept of marine fermentation was incially introduced by Zaky et al in 2014 [1,2].

References:

1 **Zaky, A. S. Marine Fermentation, the Sustainable Approach for Bioethanol Production. *EC Microbiology,* 25-27 (2017).**

2 **Zaky, A. S., Tucker, G. A., Daw, Z. Y. & Du, C. Marine yeast isolation and industrial application. *FEMS Yeast Res* 14, 813-825, doi:10.1111/1567-1364.12158 (2014).**
